# Saikosaponin-d Enhances the Anticancer Potency of TNF-**α** via Overcoming Its Undesirable Response of Activating NF-Kappa B Signalling in Cancer Cells

**DOI:** 10.1155/2013/745295

**Published:** 2013-03-12

**Authors:** Vincent Kam Wai Wong, Molly Miao Zhang, Hua Zhou, Kelly Yin Ching Lam, Po Ling Chan, Carmen Ka Man Law, Patrick Ying Kit Yue, Liang Liu

**Affiliations:** ^1^State Key Laboratory of Quality Research in Chinese Medicine, Macau University of Science and Technology, Avenida Wai Long, Taipa, Macau, China; ^2^Shum Yiu Foon Shum Bik Chuen Memorial Centre for Cancer and Inflammation Research, School of Chinese Medicine, Hong Kong Baptist University, 7 Baptist University Road, Kowloon Tong, Hong Kong; ^3^Department of Biology, Hong Kong Baptist University, Hong Kong

## Abstract

Tumor necrosis factor-alpha (TNF-**α**) was reported as anticancer therapy due to its cytotoxic effect against an array of tumor cells. However, its undesirable responses of TNF-**α** on activating NF-**κ**B signaling and pro-metastatic property limit its clinical application in treating cancers. Therefore, sensitizing agents capable of overcoming this undesirable effect must be valuable for facilitating the usage of TNF-**α**-mediated apoptosis therapy for cancer patients. Previously, saikosaponin-d (Ssd), a triterpene saponin derived from the medicinal plant, *Bupleurum falcatum* L. (Umbelliferae), showed to exhibit a variety of pharmacological activities such as antiinflammation, antibacteria, antivirus and anticancer. Recently, we found that Ssd could inhibit the activated T lymphocytes via suppression of NF-**κ**B, NF-AT and AP-1 signaling. Here, we showed that Ssd significantly potentiated TNF-**α**-mediated cell death in HeLa and HepG2 cancer cells via suppression of TNF-**α**-induced NF-**κ**B activation and its target genes expression involving cancer cell proliferation, invasion, angiogenesis and survival. Also, Ssd revealed a significant potency of abolishing TNF-**α**-induced cancer cell invasion and angiogenesis in HUVECs while inducing apoptosis via enhancing the loss of mitochondrial membrane potential in HeLa cells. Collectively, these findings indicate that Ssd has a significant potential to be developed as a combined adjuvant remedy with TNF-**α** for cancer patients.

## 1. Introduction

 Tumor necrosis factor-alpha (TNF-*α*) is initially discovered as an antitumor factor because of its remarkable ability to induce apoptosis in human malignant cells while leaving normal cells unscathed [1]. Upon binding of its cellular TNF-*α* receptor 1 (TNFR1), it induces apoptosis of cancer cells by either recruitment of TNFR-associated death domain (TRADD), and Fas-associated protein with death domain (FADD) [2, 3] or activation of reactive oxygen species (ROS) and cytochrome c release from mitochondria [4]. In addition, its significant anti-tumor effect was confirmed by injection of recombinant TNF-*α* into tumor-bearing mice [5–7]. Recently, TNF-*α* is clinically used in the treatment of soft tissue sarcoma and melanomas with an acceptable safety profile [8–10]. However, despite the profound cytotoxic and cytostatic effects of TNF-*α* on primary tumors, its undesirable effect of activating NF-*κ*B signaling and pro-metastatic nature, in particular, limit its use widely for cancer patients [11]. 

 Pathologically, TNF-*α* acts as a proinflammatory cytokine that plays an important role in inflammation and cancer, including cellular differentiation, proliferation, and apoptosis [12]; while its tumorigenic activity is mediated through activation of the pro-inflammatory transcriptional factor NF-*κ*B which induces the expression of genes linked to cancer progression and development, such as (i) antiapoptotic genes (Bcl-2, Bcl-xL, XIAP, FLIP, Survivin, and c-IAP1/2); (ii) invasive genes (MMP-9, uPA, ICAM-1, ELAM-1, and VCAM-1); (iii) growth factors (TNF-*α*, COX-2, Cyclin D1, c-MYC, interleukin 1, and interleukin 6); (iv) angiogenic factors (COX-2 and VEGF) [13, 14]. Besides, several tumor cell types such as ovarian and breast cancers were found to constitutively express TNF-*α* [15], which leads to constitutive activation of NF-*κ*B signaling, thereby rendering the tumors cell survival highly addicted to this factor [16]. Furthermore, the activation of NF-*κ*B signaling has been evident of controlling both the preneoplastic and malignant cells to resist apoptosis-based tumor-surveillance mechanisms [17]. In light of this, identification of sensitizing agents capable of suppression of TNF-*α*-induced NF-*κ*B signaling could be an attractive arena to facilitate the enhancement of TNF-*α*-mediated apoptosis for anticancer treatment.

 Saikosaponin-d (Ssd) is one of the major triterpenoid saponins derived from *Bupleurum falcatum *L. (Umbelliferae), which is commonly prescribed for inflammatory and infectious diseases by Chinese and Japanese medical doctors, while its active component Ssd was reported to have immune-modulatory, anti-inflammatory, antibacterial, antiviral, and anticancer activities [18]. Recently, we have identified that saikosaponin-d (Ssd) could exhibit antiproliferative effect on the activated T lymphocyte via suppression of NF-*κ*B, NF-AT, and AP-1 signaling [19]. 

 In the current study, we reported for the first time on how Ssd exhibits its anti-cancer effect through sensitizing TNF-*α*-induced cell death and suppressing TNF-*α*-induced NF-*κ*B activation. Besides, we found that Ssd could also inhibit TNF-*α*-induced NF-*κ*B targeted genes expression for cancer cell proliferation, invasion, angiogenesis, and survival, and abolish TNF-*α*-induced cancer cell invasion, blockage of HUVEC angiogenesis, and induce apoptosis via loss of the mitochondrial membrane potential. 

## 2. Materials and Methods

### 2.1. Chemicals, Antibodies, and Plasmid DNA

Saikosaponin-d (>98% purity, HPLC) was obtained from China Chengdu Biotechnology Company Ltd. All other chemicals were purchased from Sigma-Aldrich (St. Louis, MO, USA) unless otherwise stated. TNF-*α* was purchased from Calbiochem (San Diego, CA). Primary antibodies against P-p65^Ser536^ and Bcl-xL were purchased from Cell Signaling. Antibodies against Bcl-2, survivin, c-IAP2, XIAP, MMP-9, ICAM-1, c-myc, cyclin D1, VEGF, I*κ*B*α*, NF-*κ*B p65, caspase 3, caspase 7, caspase 9, and *β*-actin antibodies were purchased from Santa Cruz Biotechnology (Santa Cruz, CA, USA), while the COX2 antibody was purchased from Neo Markers (San Diego, CA, USA). Constitutively active IKK-beta construct was obtained from Add gene (Cambridge, MA, USA).

### 2.2. Cell Culture, Cytotoxicity Assay, and Transfection Assay

 All cells were obtained from the American Type Culture Collection (Rockville, MD, USA) unless otherwise stated. The human liver cancer cell line HepG2, human cervix cancer cell line HeLa, and human normal lung fibroblast CCD19Lu were maintained in MEM, while the human lung carcinoma cell line H1299 was maintained in DMEM. All mediums supplemented with 10% fetal bovine serum (FBS) and antibodies with 50 U/mL penicillin and 50 *μ*g/mL streptomycin (Invitrogen, Paisley, Scotland, UK). Human umbilical vein endothelial cells (HUVECs) were purchased from Clonetics (TCS Biologicals, UK) and cultured in M199 medium supplemented with 20% FCS and endothelial cell growth supplement (ECGS), heparin (Sigma, St. Louis MO, USA). All cell cultures were incubated at 37°C in a 5% humidified CO_2_ incubator. For cell cytotoxicity assay, Ssd was dissolved in DMSO at final concentration of 100 mM. Cytotoxicity was assessed using the MTT, 3-(4,5-dimethylthiazol-2-yl)-2,5-diphenyltetrazolium bromide assay (Sigma, St. Louis, MO, USA). Cells were seeded on 96-well plates (HepG2, 8000 cells; HeLa, 4000 cells; CCD19Lu, 3000 cells; H1299, 4000 cells per well). After overnight incubation, the cells were exposed to serial concentrations of Ssd (0.19–100 *μ*M) for 3 days. Subsequently, 10 *μ*L of MTT reagents was added to each well and incubated at 37°C for 4 h followed by the addition of 100 *μ*L solubilization buffer (10% SDS in 0.01 mol/L HCl) and overnight incubation. *A*_585nm_ was determined from each well the next day. The percentage of viable cells was calculated using the following formula: Cell viability (%) = Cells  number_treated_/Cells  number_untreated  control_ × 100. Data were obtained from three independent experiments. 

Cells transfection assay was performed with LTX PLUS reagent (Invitrogen); HepG2 cells (4 × 10^5^ cells/well) were seeded in 35 mm dish before the day of transfection. Next day, 100 *μ*L of Opti-MEM was mixed with 1 *μ*g of constitutively active IKK-*β* plasmid DNA together with 3 *μ*L of PLUS reagent at room temperature for 5 min. After that, 3 *μ*L of LTX transfection reagent was then added into the mixture with gentle stirring by pipette tip and stood at room temperature for another 30 min. Meanwhile, the cells were changed with fresh medium without antibiotic. Finally, 100 *μ*L of Opti-MEM/LTX PLUS reagent/constitutive IKK-*β* plasmid mixture was added dropwise onto the cells. The transfected cells were cultured in 37°C incubator for at least 24 h and then suggested to cell seeding for MTT assay. 

### 2.3. Western Blot Analysis

 Cancer cells (5–7 × 10^5^) were preincubated with different concentrations of Ssd at a range of 7.5–15 *μ*M for 60 min and then treated with 20 ng/mL of TNF-*α*. The total cell extracts were then harvested after 15 min for Western blotting analysis. For NF-*κ*B p65 nuclear translocation detection, subcellular proteins from cytosolic and nuclear fractions of the treated cancer cells were extracted using NucBuster Protein Extraction Kit (Novagen). The fractionated cytosolic proteins (50 *μ*g) and nuclear extracts (30 *μ*g) were resolved by 10% SDS/PAGE. After electrophoresis, the proteins were electrotransferred to nitrocellulose membrane which was then blocked with 5% dried milk for 60 min. The membrane was then washed three times for 5 min each with wash buffer and incubated with corresponding primary antibodies overnight at 4°C. Following washing, the membrane was further incubated with HRP-conjugated secondary antibodies for 60 min. The blot was developed using the ECL Western Blotting Detection Reagents (Amersham Biosciences). 

### 2.4. Immunocytochemistry Assay

HeLa cells were grown on glass coverslips in six-well plates (35 mm diameter). After exposure to 10 *μ*M Ssd and 20 ng/mL TNF-*α*, cells were washed once with PBS and then fixed with 4% paraformaldehyde in PBS for 15 min. The fixed cells were washed thrice with PBS and then permeabilized with 0.1% Triton X-100 in PBS for 5 min. The cells were then washed with PBS for two times and then stained with DAPI (Sigma) for 15 min to visualize the cell nucleus. For NF-*κ*B p65 staining, cells were then incubated with p65 antibody (Santa Cruz) (1 : 200) in 3% bovine serum albumin for 2 h. FITC-conjugated goat anti-rabbit IgG (ZYMED, San Francisco, CA, USA) (1 : 500) antibody was used as the secondary antibody for another 1 h. Excess antibody was removed by multiple washes with TBS. Coverslips were mounted onto microscope slides with FluorSave Reagent (Calbiochem) and examined by widefield epifluorescence microscopy. Images were captured by a Photometrics CoolSNAP HQ^2^ CCD camera on the Olympus IX71-Applied Precision DeltaVision restoration microscope, and the epifluorescence images were matched using DeltaVision algorithms (Applied Precision, Inc., Applied Precision DeltaVision Elite). 

### 2.5. Cell Invasion Assay

The cancer cell invasion assay was performed in a cell invasion chamber, a 24-well tissue culture plate with cell culture inserts that contain an 8 *μ*m pore size polycarbonate membrane over a thin layer of dried ECMatrix (CHEMICON). H1299 cells (15000 cells/well) were resuspended in serum-free medium and incubated in an invasion chamber insert with different concentrations of Ssd with or without 20 ng/mL TNF-*α* for 72 h, while the lower chamber contained medium with 10% FBS. The cells invaded through the ECM layer to the bottom of the polycarbonate membrane were labeled with Cell Stain provided in the kit for 20 min at room temperature. The non-invading cells were gently removed from the interior of the inserts by using a cotton-tipped swab. The number of invaded cells was counted through the microscope and quantified by dissolving stained cells in 10% acetic acid (200 *μ*L/well). The colorimetric reading of the solute mixture was determined by spectrophotometer at OD 560 nm.

### 2.6. Tube Formation Assay

96-well plates were coated with 60 *μ*L of growth factor-reduced Matrigel (BD Biosciences, Bedford, MA, USA) and incubated at 37°C for 30 min. HUVECs treated 0.5% FBS EGM medium for 24 h were treated with trypsin-EDTA and suspended in the culture medium. The HUVECs were incubated with different concentrations of Ssd for 30 min at room temperature before seeding. The Ssd-treated HUVECs (2 × 10^4^ cells/well) were seeded on polymerized Matrigel in the presence of VEGF (10 ng/mL). After incubation at 37°C for 8 h, photographs were taken using Motic Image Plus 2.0 software (Motic Instruments Inc., Canada). The angiogenic activity of HUVEC under different treatments was determined by counting the branch points of the tubes formed in the well. Three independent experiments were performed, and each experiment was run in triplicate. 

### 2.7. Cell Migration Assay

HUVECs (3 × 10^4^ cells/well) were plated onto gelatin (0.1%) precoated 96-well plates and incubated for 24 h. An artificial wound was created by using mechanical scratching of the cell monolayer [20]. The denuded area in each well was captured using Motic Image Plus 2.0 software (Motic Instruments Inc., Canada). Cells were then incubated for further 16 or 24 h with the presence of 5 or 10 *μ*M Ssd and the denuded area of each was captured again. Images at 0, 16, and 24 h were analyzed using Java's Image J software. The migration of cells towards the wounds was expressed as percentage of recovery:

(1) $$\begin{array}{c} \%\;\text{of}\;\text{recovery}=\left[\frac{At=0-At=\text{end}\;\text{point}}{At=0}\right]\times 100\%, \end{array}$$

where *At* = 0 is the area of wound measured immediately after scratching, and *At* = end-point is the area of wound measured 16 or 24 h after scratching.

### 2.8. Analysis of Mitochondrial Membrane Potential

Drug-treated HeLa cells were analyzed for its mitochondrial membrane potential (ΔΨm) using JC-1 dye (Life Technologies) according to the manufacturer's instructions. In brief, HeLa cells were first treated with 10 *μ*M of Ssd with or without TNF-*α* for 4 h; the cells were then stained with JC-1 at 2 *μ*M at 37°C, 5% CO_2_, for 30 min. Subsequently, the cells were washed with PBS prior to fluorescence imaging using Photometrics CoolSNAP HQ^2^ CCD camera on the Olympus IX71-Applied Precision DeltaVision restoration microscope (Applied Precision DeltaVision Elite). Orange-fluorescent signal indicates the cells with hyperpolarized membrane potentials, while green-fluorescent signal indicates the cells with depolarized membrane potentials.

### 2.9. LIVE/DEAD Viability/Cytotoxicity Assay

 Drug-treated HeLa and HepG2 cancer cells were analyzed for its cell viability using LIVE/DEAD Cell Imaging Kit (Life Technologies) according to the manufacturer's instructions. In brief, HeLa and HepG2 cancer cells were first treated with 10 *μ*M of Ssd with or without TNF-*α* for 24 h; the Live Green and Dead Red reagents were then mixed to prepare 2X stock and added to the cells at room temperature for 15 min. The cells were subjected to fluorescence imaging using Photometrics CoolSNAP HQ2 CCD camera on the Olympus IX71-Applied Precision DeltaVision restoration microscope (Applied Precision DeltaVision Elite). Cells with green fluorescence signal represent live cells, while cells with red fluorescence signal represent dead cells. 

### 2.10. Statistical Analysis

 The data are expressed as means ± SD as indicated. The difference was considered statistically significant when the *P* value was less than 0.05. Student's *t*-test or one-way ANOVA analysis was used for comparison among different groups.

## 3. Results

### 3.1. Ssd Significantly Sensitizes TNF-*α*-Mediated Cell Death in Cancer Cells via Disturbance of Mitochondrial Membrane Potential (ΔΨm)

**Figure 1:**
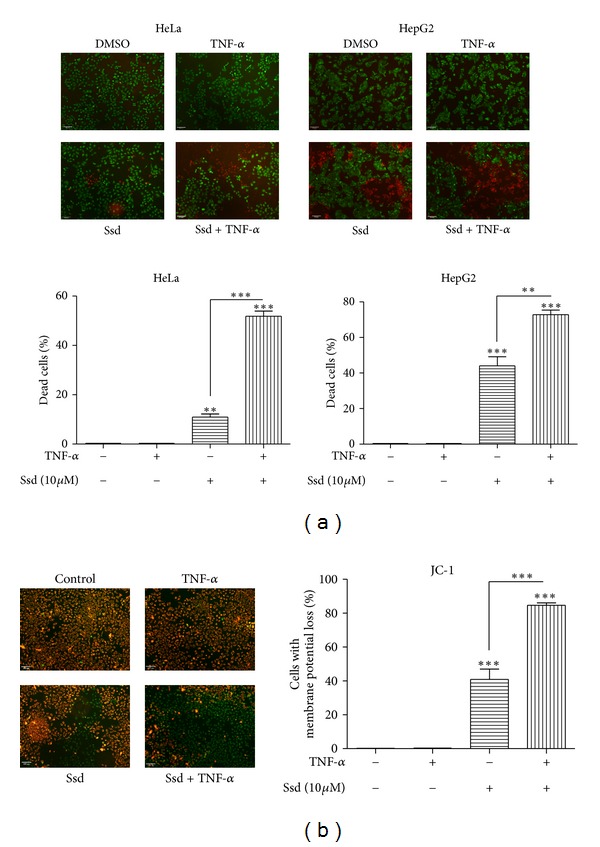
Inducing effect of Ssd on the TNF-*α*-mediated cell death through potentiating the loss of mitochondrial membrane potential (ΔΨm). (a) Ssd increases the percentage of cancer cell death in the presence of TNF-*α*. HeLa and HepG2 cancer cells were treated with 10 *μ*M of Ssd with or without 20 ng/mL TNF-*α* for 24 h. The LIVE/DEAD Cell Imaging Kit (Life Technologies) reagents were mixed and added to the cells for 15 min. The cells were subjected to fluorescence imaging using Olympus IX71-Applied Precision DeltaVision restoration microscope. Cells with green fluorescence signal represent live cells, while cells with red fluorescence signal represent dead cells. The percentage of dead cells was quantified for HeLa and HepG2 and indicated in bar chart. (b) Ssd enhances the (ΔΨm) loss in the presence of TNF-*α*. HeLa cells were treated with 10 *μ*M of Ssd with or without 20 ng/mL TNF-*α* for 4 h, the cells were then stained with 2 *μ*M of JC-1 for 30 min. Fluorescence images were captured using Olympus IX71-Applied Precision DeltaVision restoration microscope and the percentage of cells with (ΔΨm) loss was quantified and indicated in bar chart. Orange-fluorescent signal indicates the cells with hyperpolarized membrane potentials, while green-fluorescent signal indicates the cells with depolarized membrane potentials. **P < 0.01 and ***P < 0.001. Three independent experiments were performed, and more than 5000 cells were scored from 5 different views of captured images.

Although TNF-*α* has been evident of suppressing cancerous carcinoma, it also stimulates activation of NF-*κ*B signaling via degradation of I*κ*B*α*, leading to apoptosis resistance, as well as TNF-*α* resistance in cancer cells [21]. We previously reported that Ssd inhibits NF-*κ*B activation induced by PMA/Ionomycin in mouse T lymphocytes [19]. However, the function of Ssd on TNF-*α*-induced apoptosis in cancer cells is not fully understood. To address this question, two TNF-*α*-resistant cancer cell types from different origin [22, 23], that is, human cervical carcinoma (HeLa) and human liver cancer cells (HepG2), were used to demonstrate the general sensitizing effect of Ssd on TNF-*α*-mediated cell death in cancer cells; we performed the LIVE/DEAD Viability assay to discriminate live from dead cells by simultaneously staining with green-fluorescent calcein-AM to indicate intracellular esterase activity and red-fluorescent ethidium homodimer-1 to indicate the loss of plasma membrane integrity that analyzed the effects of combined treatment with Ssd and TNF-*α* in HeLa and HepG2 cancer cells. As shown in Figure 1(a), treatment with DMSO control and TNF-*α* (20 ng/mL) alone indicated no significant cell death (red-fluorescent) in both cancer cell types. However, treatment with Ssd (10 *μ*M) alone exhibited a certain effect on cell viability by presenting the increased number of cells with red-fluorescent signal. Notably, combination of Ssd with TNF-*α* resulted in a significant increase in cell death by demonstrating over 50% and 70% of cells with red-fluorescent signal in HeLa and HepG2 cells, respectively (Figure 1(a) lower panel, bar chart). In order to investigate whether apoptosis is involved upon the treatment of Ssd and TNF-*α*, we determined the mitochondrial membrane potential (ΔΨm) in HeLa cancer cells using live-cells imaging with JC-1 dye. The membrane-permeable JC-1 dye is widely used in apoptosis studies to monitor mitochondrial health. JC-1 dye exhibits potential-dependent accumulation in mitochondria, indicated by a fluorescence emission shift from green (~529 nm) to orange (~590 nm). Consequently, mitochondrial depolarization (i.e., apoptosis) is indicated by a decrease in the orange/green fluorescence intensity ratio [24]. As shown in Figure 1(b), treatment with DMSO control and TNF-*α* (20 ng/mL) alone showed no significant damage to mitochondrial health (orange fluorescence) in HeLa cells. However, treatment with Ssd (10 *μ*M) alone demonstrated ~40% of cells with the loss of (ΔΨm) by decreasing in the orange/green fluorescence intensity ratio. Obviously, combination of Ssd with TNF-*α* resulted in more than 80% of cells with green-fluorescent intensity, suggesting that the cells undergo mitochondrial depolarization and early apoptosis (Figure 1(b), right panel). Collectively, Ssd was found to have an additive or synergistic effect with TNF-*α* for inducing cell death in both HeLa and HepG2 cancer cells. 

### 3.2. Ssd Attenuates TNF-*α*-Induced NF-*κ*B Activation

**Figure 2:**
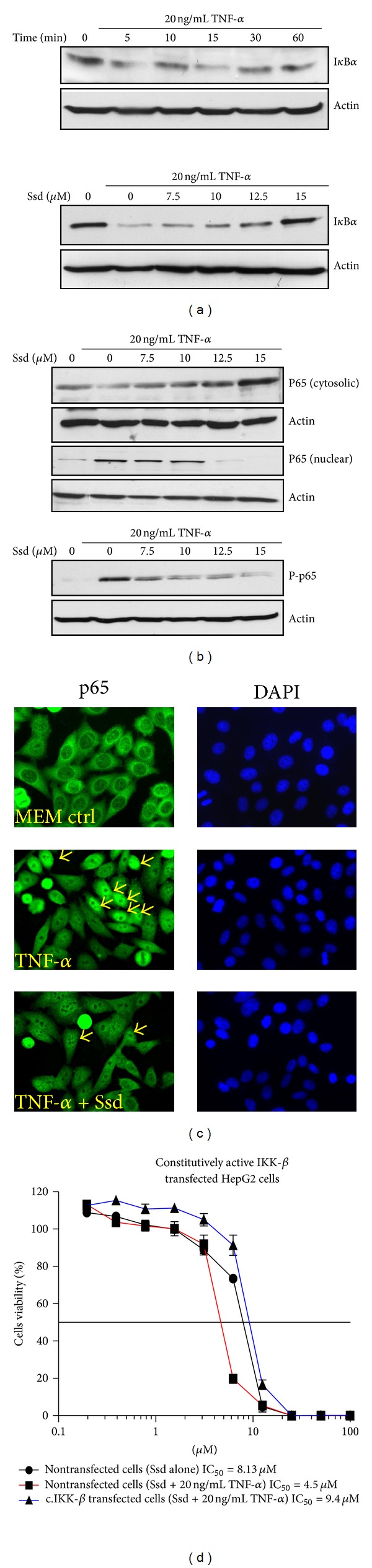
Suppressive effect of Ssd on the TNF-*α*-induced NF-*κ*B signaling. (a) Effect of Ssd on TNF-*α*-induced degradation of I*κ*B*α*. Upper panel, TNF-*α*-induced the degradation of I*κ*B*α*; lower panel, Ssd inhibited TNF-*α* induced degradation of I*κ*B*α*. HepG2 cells were pretreated with indicated concentrations of Ssd for 60 min and then incubated with 20 ng/mL TNF-*α* at 37°C for 15 min. The I*κ*B*α* in cytosolic extracts was detected by Western blotting. (b) Effect of Ssd on TNF-*α*-induced phosphorylation and nuclear translocation of NF-*κ*B p65. Upper panel, Ssd inhibited TNF-*α*-induced nuclear translocation of NF-*κ*B p65; lower panel, Ssd inhibited TNF-*α* induced phosphorylation of NF-*κ*B p65. HepG2 cells were preincubated with indicated concentrations of Ssd for 60 min, and then the cells were treated with 20 ng/mL TNF-*α* for 15 min. The cytosolic and nuclear extracts were harvested for detection of p65, while the total cell extracts were harvested for the detection of phosphorylated form of p65. (c) Immunocytochemical analysis of NF-*κ*B p65 nuclear translocation. HeLa cells were pretreated with 10 *μ*M Ssd for 60 min, and then cells were treated with TNF-*α* for 30 min. Cells were fixed in 4% paraformaldehyde and costained with anti-p65 antibodies (green) and DAPI (blue). Arrows indicate the cells with p65 being translocated into nucleus under magnification 400x. (d) Cytotoxicity of Ssd with or without TNF-*α* in constitutively active IKK-*β* transfected HepG2 cells. HepG2 cells were transfected with or without c.IKK-*β* plasmid being subjected to Ssd treatment in the presence or absence of TNF-*α* (20 ng/mL) for 72 h. The cell viability was determined by MTT assay. Results are mean ± SD from three independent experiments.

 Stimuli like TNF-*α* and LPS can activate the canonical NF-*κ*B pathway; then the transcriptional factor NF-*κ*B is released to the nuclear after the phosphorylation, ubiquitination, and proteolytic degradation of I*κ*B*α* [12, 17]. To examine the suppressive effect of Ssd on TNF-*α*-induced I*κ*B*α* degradation, we examined the proteolytic degradation of I*κ*B*α* in the presence of TNF-*α* from 0 to 60 min. Results showed that I*κ*B*α* began to degrade at 5 min and reached at the maximum at 15 min (Figure 2(a), upper panel). Accordingly, 15 min of TNF-*α* stimulation on HepG2 cells is optimal for inducing I*κ*B*α* degradation. Next, HepG2 cells were pretreated with indicated concentrations of Ssd for 1 h prior to 15 min stimulation with TNF-*α*. Results showed that Ssd inhibited TNF-*α*-induced I*κ*B*α* degradation in a dose-dependent manner (Figure 2(a), lower panel). Then, we further determined whether Ssd affects TNF-*α*-induced NF-*κ*B subunit and p65 nuclear translocation. Accordingly, HepG2 cells were pretreated with indicated concentrations of Ssd and then stimulated with TNF-*α* for 15 min. The cells cytosolic and nuclear extracts were then prepared for Western blotting analysis using antibody against p65. Results demonstrated that Ssd could not only increase the cytosolic fraction of NF-*κ*B p65, but also downregulate TNF-*α*-induced nuclear translocation of p65 in a dose-dependent manner (Figure 2(b) upper panel), suggesting that TNF-*α* mediated nuclear translocation of p65 is suppressed by Ssd treatment. Given that the phosphorylation of p65 is required for its transcriptional activity and subsequent translocation to the nucleus [25], we, therefore, determined the effect of Ssd on TNF-*α*-induced p65 phosphorylation on HepG2 cells. As expected, Ssd was shown to suppress TNF-*α* induced phosphorylation of p65 in a dose-dependent manner (Figure 2(b), lower panel). Furthermore, we also visualized the suppressive effect of Ssd on TNF-*α*-induced NF-*κ*B p65 nuclear translocation by immunocytochemistry with HeLa cells. HeLa is a human cancer cell line being widely adopted for immunocytochemistry study because it provides a discrete compartment for immunofluorescence imaging analysis [26]. For this purpose, HeLa cells were pretreated with 10 *μ*M Ssd for 1 h and then stimulated with TNF-*α* for 30 min. The nuclear translocation of NF-*κ*B p65 was detected by antibody against p65. Results demonstrated that there was no p65 expression in the nuclear region of the untreated control cells. However, addition of TNF-*α* strongly stimulated nuclear translocation of p65 in HeLa cells and this nuclear translocation was inhibited by Ssd treatment (Figure 2(c)). Taken together, these results suggest that Ssd has potent inhibitory effect on the TNF-*α*-induced phosphorylation and nuclear translocation of NF-*κ*B p65.

### 3.3. The Constitutively Activated IKK-*β*-NF-*κ*B Signaling Abrogates the Additive Effect of TNF-*α* and Ssd in the Ssd-Mediated Cell Cytotoxicity in HepG2 Cells

It seems that the tumor suppression effect with Ssd in combination of TNF-*α* is probably contributed by downregulation of NF-*κ*B signaling. To further verify whether activation of NF-*κ*B signaling would diminish the enhancing effect of TNF-*α* in Ssd-mediated cell cytotoxicity, we specifically activated the NF-*κ*B signaling by overexpression of the constitutively activated IKK-*β* (c.IKK-*β*) in HepG2 cells and saw if the cell viability could be recovered from the Ssd-TNF-*α* treatment. In nontransfected HepG2 cells, the mean IC_50_ of Ssd is 8.13 *μ*M; addition of TNF-*α* would further enhance the Ssd-mediated cell cytotoxicity with mean IC_50_ = 4.5 *μ*M. However, constitutively activation of IKK-*β*-NF-*κ*B signaling in HepG2 cells abrogated the potentiating effect of TNF-*α* in Ssd-mediated cell cytotoxicity with mean IC_50_ = 9.4 *μ*M (Figure 2(d)). Collectively, the results suggest that the Ssd-mediated cell cytotoxicity in the presence of TNF-*α* is manifested by the modulation of NF-*κ*B signaling.

### 3.4. Ssd Counteracts TNF-*α*-Induced NF-*κ*B-Dependent Genes Expression in HeLa and HepG2 Cells

**Figure 3:**
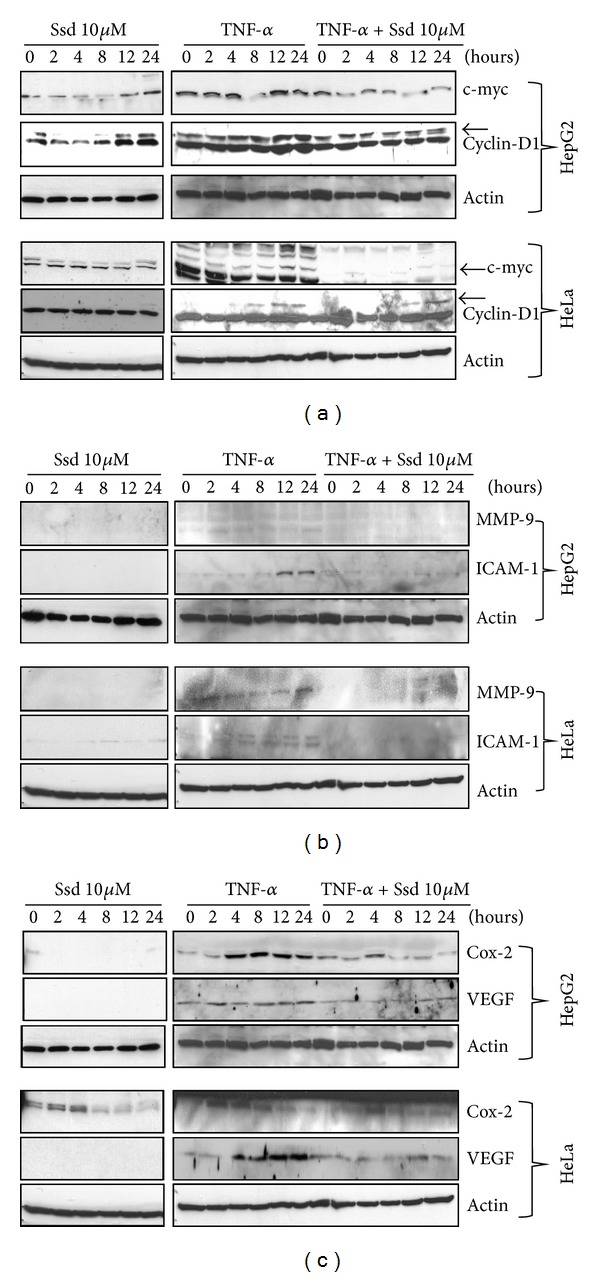
Down-regulatory effect of Ssd on the TNF-*α*-induced NF-*κ*B-dependent genes expression. (a) Ssd downregulates TNF-*α*-induced NF-*κ*B dependent cell proliferative genes expression. (b) Ssd downregulates TNF-*α*-induced NF-*κ*B-dependent invasive genes expression. (c) Ssd downregulates TNF-*α*-induced NF-*κ*B-dependent angiogenic genes expression. HepG2 and HeLa cells were pretreated with 20 ng/mL TNF-*α* for 60 min and then subjected to 10 *μ*M of Ssd treatment as indicated in time intervals, and the whole cell extracts were prepared and analyzed by Western blotting. Gel images are representative of three independent experiments.

Although TNF-*α* exhibits suppressive effect in various types of cancer cells, the emerging evidences suggest that TNF-*α* would activate NF-*κ*B signaling, in turn to initiate the NF-*κ*B targeted genes expression which may potentiate the cell proliferation, invasion, and angiogenesis [27]. For instance, NF-*κ*B activation induces cell proliferation and cell-cycle progression by regulating the expression of the targeted genes including growth factors such as IL-2 and cell-cycle regulators of c-myc and cyclin D1. The question comes if Ssd exhibits any effect on expression of c-myc and cyclin D1 in response to TNF-*α* treatment. Accordingly, HepG2 and HeLa were treated with TNF-*α* in the presence or absence of 10 *μ*M Ssd for the indicated periods of time (0–24 h) and then examined the NF-*κ*B-dependent expression of c-myc and cyclin D1 by Western blotting analysis. Results showed that Ssd treatment alone would not significantly induce the expression of c-myc and cyclin D1, while these genes expression was markedly upregulated upon TNF-*α* treatment. However, the treatment of Ssd could effectively suppress the TNF-*α*-mediated expression of these NF-*κ*B targeted genes in both HeLa and HepG2 cancer cells (Figure 3(a)). In addition, the matrix metalloproteinases, cyclooxygenases, and adhesion molecules are the target genes of NF-*κ*B signaling playing a crucial role in facilitating tumor cell invasion of the extracellular matrix [28], while TNF-*α* has been evident in inducing expression of genes involving tumor metastasis such as MMP-9 and ICAM-1 [29]. As expected, Ssd treatment alone would not induce the expression of MMP-9 and ICAM-1. However, the expression of MMP-9 and ICAM-1 was increased in response to TNF-*α* stimulation, while the addition of Ssd abrogated the TNF-*α*-mediated expression of these NF-*κ*B target genes (Figure 3(b)). Furthermore, angiogenesis is the process whereby endothelial cells build new blood vessels from existing vasculature. Inhibiting angiogenesis may be an effective way to inhibit tumor growth and metastasis [30]. As mentioned before, VEGF and COX-2 are the essential genes for angiogenesis of tumors [31], and these genes are always upregulated in response to TNF-*α*-activated NF-*κ*B signaling [13, 14]. The results showed that Ssd alone would not induce the expression of COX-2 and VEGF in both cancer cell types; however, these genes expression was significantly increased in response to TNF-*α* treatment, while treatment of Ssd was able to inhibit the TNF-*α*-mediated expression of COX-2 and VEGF (Figure 3(c)). Taken together, Ssd could effectively suppress the TNF-*α*-induced NF-*κ*B-dependent genes expression and then inhibit cancer cell proliferation, invasion, and angiogenesis.

### 3.5. Ssd Abates TNF-*α*-Mediated NF-*κ*B Dependent Antiapoptotic Genes Expression and Activates the Cleavage of Apoptotic Mediators

**Figure 4:**
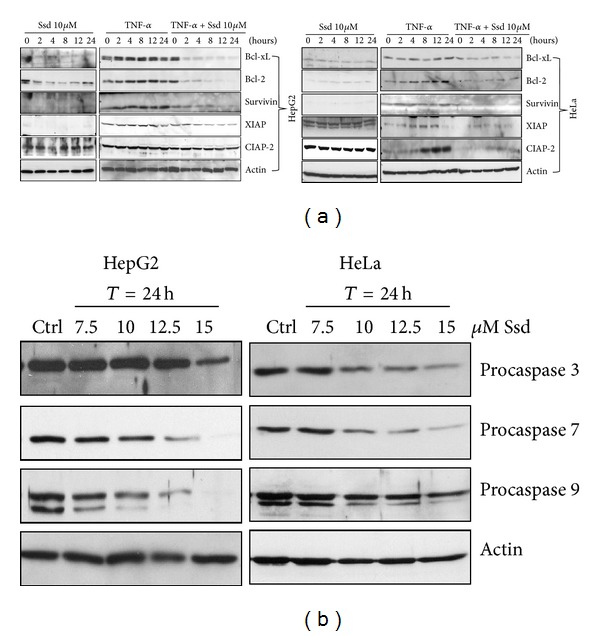
Diminishing effect of Ssd on the TNF-*α*-inducted antiapoptotic genes expression. (a) Ssd abates TNF-*α*-mediated NF-*κ*B-dependent anti-apoptotic genes expression in cancer cells. HepG2 and HeLa cells were pretreated with 20 ng/mL TNF-*α* for 60 min and then subjected to 10 *μ*M of Ssd treatment for indicated time intervals, and the whole cell extracts were prepared and analyzed by Western blotting. (b) Ssd induces the cleavage of procaspases in cancer cells. HepG2 and HeLa cells were treated with indicated concentrations of Ssd (7.5, 10, 12.5 or 15 *μ*M) for 24 h. The whole cell extracts were then prepared analyzed by Western blot using antibodies against procaspases 3, 7, and 9; while actin was used as loading control. Gel images are representative of three independent experiments.

Given that TNF-*α* is found to activate NF-*κ*B signaling which is followed by the upregulation of NF-*κ*B target genes expression involving antiapoptosis [27], overexpression of the NF-*κ*B-dependent antiapoptotic proteins such as Bcl-2 or Bcl-xL would block the release of apoptotic regulator, cytochrome c in turn to suppress apoptotic stimuli. In addition, the IAP-related genes provide very potent protection against proapoptotic stimuli. For instance, the antiapoptotic activity of XIAP is attributed by its ability to bind and suppress caspases [32–34]. Besides, survivin appears to modulate apoptotic signaling through the control of cell cycle and cell proliferation, as well as destabilization of p53 tumor suppressor [35, 36]. Other anti-apoptotic proteins like c-IAP1 and c-IAP2 being recruited to TNFR-1 and -2 signaling complexes can modulate the activity of caspase 8 through TRAF2 binding [37, 38]. Furthermore, another anti-apoptotic protein FLIP can regulate the cell surface death receptor activation of caspase through resembling to caspase 8 [39] or expression of decoy receptors for TRAIL [40]. Since Ssd could effectively suppress TNF-*α* induced NF-*κ*B signaling, we further investigated whether Ssd treatment could affect the NF-*κ*B-dependent genes expression, particularly related to antiapoptosis. The results demonstrated that Ssd treatment alone exhibited no significant activation of anti-apoptotic genes expression, while TNF-*α*-induced expression of NF-*κ*B target genes including Bcl-2, Bcl-xL, XIAP, c-IAP2, and survivin was markedly suppressed by Ssd in a time-dependent manner (Figure 4(a)). Apart from studying the suppression effect of Ssd on TNF-NF-*κ*B-related anti-apoptotic genes expression, it is interesting to know whether Ssd alone would activate apoptotic mediators, leading to apoptosis in cancer cells. As caspases 3, 7, and 9 are important mediators of apoptosis and contribute to the overall apoptotic morphology by cleavage of various cellular substrates [41], we, therefore, investigated the role of these caspases in Ssd-mediated apoptotic cell death in both HepG2 cells and HeLa cells. Results indicated that Ssd could activate the cleavage of the procaspase-3, 7, and 9 in both cell types in a dose-dependent manner (Figure 4(b)). These results suggest that Ssd contributes to apoptotic cell death, at least in part, through the suppression of TNF-NF-*κ*B-mediated anti-apoptotic genes expression and direct activation of apoptotic mediators such as caspases 3, 7 and 9.

### 3.6. Ssd Reverses TNF-*α*-Induced Cell Invasion

**Figure 5:**
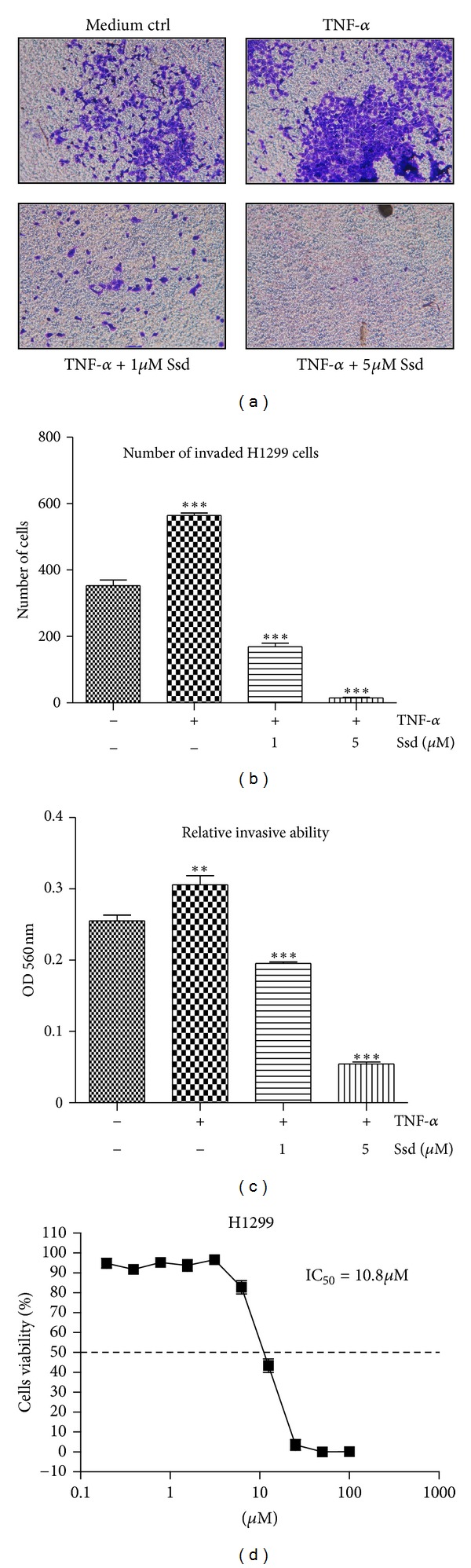
Inhibitory effect of Ssd on the TNF-*α*-induced cancer cell invasion. (a) Ssd suppresses the TNF-*α*-mediated cell invasion in H1299 cells. Images of invasive cells found in the lower layer of ECMatrix chamber were captured by digital camera under microscope with 100X magnification. (b) The number of invasive cells was counted in the lower layer of ECMatrix chamber membrane. ***P < 0.001 for comparison between medium control and TNF-*α* treatment alone and for Ssd treatment compared to TNF-*α* treatment alone. (c) The absorbance reading at OD 560 nm of the stained invasive cells solute. **P < 0.01 and ***P < 0.001 compared to medium control and TNF-*α* treatment alone, respectively. (d) Cytotoxicity of Ssd on H1299 cells. Results are mean ± SD from three independent experiments.

Apart from examining the effect of Ssd on TNF-*α*-induced NF-*κ*B-dependent genes expression involving cell invasion, we further determined whether Ssd could modulate the tumor cell invasion activity induced by TNF-*αin vitro*. For this purpose, we firstly investigated which cancer cell lines contain strong invasive activity in the presence of TNF-*α*. Our preliminary data indicated that both HeLa and HepG2 cells exhibited a very low invasive ability, while H1299 cells revealed a significant invasive ability in the presence of TNF-*α* that is acceptable as a cellular model for performing cell invasion assay [42]. Accordingly, H1299 cells were adopted in the invasion assay of current studies and seeded into the insert of an ECMatrix invasion chamber in the absence of serum. Meanwhile, H1299 cells were incubated with different concentrations of Ssd in the presence or absence of TNF-*α*. As shown in Figure 5(a), H1299 cells were found to exhibit certain extent of cell invasion ability in the absence of TNF-*α*, while the number of invaded cells was significantly increased in the presence of TNF-*α*. However, the addition of Ssd could dose-dependently reduce the number of invaded cells even in the presence of TNF-*α*. To quantify the cell invasion activity in response to Ssd treatment, a number of invaded H1299 cells found in the lower layer of the ECMatrix chamber were stained and counted (Figure 5(b)). The amount of staining dye accumulated in the stained cells was further quantified with spectrophotometer at OD560 nm (Figure 5(c)). Taken together, Ssd was found to suppress TNF-*α*-induced cell invasion activity in dose-dependent manner. Besides, the effective dose of Ssd in the suppression of cell invasion activity is between 1 and 5 *μ*M, which far below the mean IC_50_ (10.8 *μ*M) of Ssd in cytotoxicity assay of H1299 cells (Figure 5(d)), suggesting that the Ssd-mediated suppression of cell invasion activity is specific. 

### 3.7. Ssd Suppresses the HUVEC Tube Formation and Migration *In Vitro*

**Figure 6:**
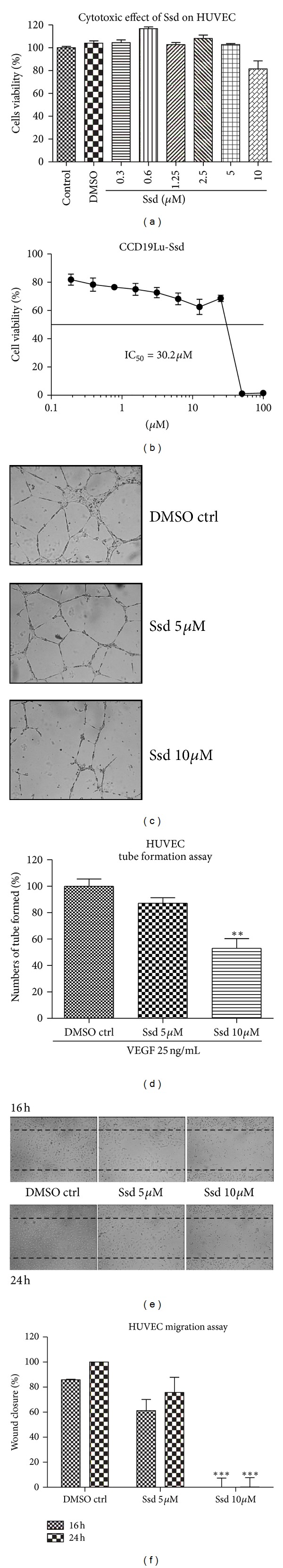
Suppressive effect of Ssd on the HUVEC's tube formation and cell migration. (a) Cytotoxicity assay of Ssd in HUVECs. (b) Cytotoxicity assay of Ssd in CCD19Lu. (c) Ssd inhibits the formation of endothelial cells (EC) tube networks in HUVECs. The HUVECs were incubated with different concentrations of Ssd for 30 min at room temperature before seeding. DMSO or Ssd-treated HUVECs were seeded on polymerized Matrigel in the presence of VEGF; the images of EC tube networks were captured after incubation for 8 h at 37°C. (d) The numbers of the tube formed were quantified under light microscope. (e) Ssd inhibits the cell migration in HUVECs. The HUVECs were incubated onto gelatin-coated plates for 24 h. An artificial wound was created and the denuded area in each well was captured using Motic Image Plus microscope. HUVECs were then incubated with 5 or 10 *μ*M of Ssd for further 16 or 24 h and the denuded areas were captured and analyzed using Java's Image J software. (f) The migration of cells towards the wounds was expressed as percentage of wound closure for 16 and 24 h. Results are presented from three independent experiments.

TNF-*α* is implicated in the processes of tumor growth, survival, differentiation, invasion, metastases, secretion of cytokines, and proangiogenic factors [43]. Recent studies showed that binding of TNF-*α* to distinct receptor, TNFR2/p75, promotes ischemia-induced angiogenesis via modulation of several angiogenic growth factors including VEGF [43]. Aforesaid, VEGF and COX-2 are the angiogenic growth factors to promote angiogenesis during cancer development [31]. As shown in Figure 3(c), we clearly showed that Ssd was able to inhibit the TNF-*α*-mediated expression of COX-2 and VEGF. In this connection, we directly adopted the VEGF to stimulate the angiogenic process in HUVEC and determined if Ssd could suppress tumor growth by the inhibition of VEGF-mediated angiogenesis. As shown in Figure 6(a), Ssd showed no significant cytotoxic effect on HUVECs up to 10 *μ*M, and it also demonstrated 3-folds-less toxic in human normal lung fibroblasts, CCD19Lu with mean IC_50_ (30.2 *μ*M) (Figure 6(b)). Besides, in the presence of Matrigel with VEGF, HUVECs were induced to form EC tube networks *in vit*ro (Figure 6(c)). However, 10 *μ*M of Ssd markedly inhibited the formation of EC tube networks by around 50% (Figures 6(c) and 6(d)). In addition, HUVECs migration was also monitored upon the Ssd treatment for 16 and 24 h. Results indicated that Ssd was able to suppress HUVEC's migration in a dose-dependent manner up to almost 100% inhibition by 10 *μ*M concentration of the drug (Figure 6(e)), and the percentage of HUVEC's wound closure in response to Ssd treatment was quantified in Figure 6(f). Taken together, these data suggest that Ssd may exhibit an antiangiogenic effect in tumor cells. In addition, the effective dosage of Ssd in the suppression of HUVEC's tube formation and cell migration is between 5 and 10 *μ*M which showed no significant cell cytotoxicity in HUVECs as well as human normal lung fibroblast, CCD19Lu (Figures 6(a) and 6(b)), suggesting that the antiangiogenic effect of Ssd is specific, but not due to its direct cell cytotoxicity. 

## 4. Discussion

 TNF-*α* was reported as anticancer therapy due to its cytotoxic effect against a number of tumor cells [44]. Nevertheless, clinical benefits of TNF-*α*are limited as it induces NF-*κ*B-dependent proinflammatory and antiapoptotic genes expression that causes resistance to chemotherapy in many types of tumors [12]. Sensitizing agents capable of overcoming this resistance may facilitate the establishment of TNF-*α*-mediated apoptosis remedy against cancers in clinic [45]. Moreover, the clinical chemotherapeutic agent, paclitaxel, has been shown to induce soluble TNF-*α* production in macrophages, and these alterations in tumor necrosis factor signaling pathways are associated with cytotoxicity and resistance to taxanes in tumor cells [46]. Clinically, the combined treatment of NF-*κ*B inhibitor with TNF-*α* or with chemotherapeutic agents containing NF-*κ*B-activated effect may offer a new strategy for the treatment of a variety of human cancers that are resistant to TNF-*α* or chemotherapy treatment alone. Here, we for the first time revealed that the combined treatment with Ssd and TNF-*α* could significantly potentiate cell death in HeLa and HepG2 cancer cells which are insensitive to TNF-*α* treatment alone. In addition, Ssd not only inhibited TNF-*α*-induced NF-*κ*B activation by the prevention of I*κ*B*α* degradation, NF-*κ*B p65 phosphorylation and nuclear translocation, but also downregulated TNF-*α*-induced NF-*κ*B-dependent genes expression involving cancer cell proliferation (c-myc and cyclin D1), cell invasion (MMP-9 and ICAM-1), angiogenesis (COX-2 and VEGF), and anti-apoptosis (survivin, c-IAP2, Bcl-2, Bcl-xL and XIAP), leading to induction of apoptosis, and blockage of cell invasion and angiogenesis. On the contrary, the cytotoxic effect of Ssd plus TNF-*α* on HepG2 cancer cells was abated by constitutive activation of IKK-*β*, suggesting that the anti-cancer effect of Ssd with TNF-*α* is at least in part through suppression of NF-*κ*B signaling. In addition, this result indicates that Ssd may target the upstream signaling pathway of IKK-*β*.

 The oncogenic growth factors such as cyclin D1 and c-myc are required for cancer cells to enter from G1 phase to S phase of the cell cycle, and their aberrant expression is commonly found in a variety of tumors [47, 48]. The results of Ssd on downregulating cyclin D1 and c-myc genes expression may provide better understanding of how Ssd induced G1/S cell cycle arrest reported previously [49]. In addition, the c-myc promoter is transcriptionally regulated by nuclear factor of the activated T cells (NFAT), while this NFAT signaling has been found to control cell cycle regulation, apoptosis, angiogenesis, and metastasis [50]. Concomitantly, our previous findings indicate that Ssd suppresses inflammatory response via downregulation of NFAT signaling [19]. Accordingly, Ssd-mediated suppression of c-myc expression may be not only contributed by downregulation of NF-*κ*B signaling, but also compromised by suppression of NFAT signaling in cancer cells. 

 Cell invasion through the extracellular matrix is a vital step in tumor metastasis. Here, we showed that the tumor cell invasive genes (e.g., MMP-9 and ICAM-1) were all suppressed by Ssd treatment. MMP-9 plays a crucial role in tumor invasion and angiogenesis by mediating degradation of the extracellular matrix [51], and inhibition of MMP-9 activity has been shown to suppress nonsmall-cell lung cancer (H1299) metastasis in nude mice xenografts [52], while ICAM-1 activation is found to contribute migration of human lung cancer cells [53]. However, recent studies reported a controversial role of ICAM-1, that is, its activation is necessary to induce the tissue inhibitor of matrix metalloproteinases-1 (TIMP-1) and a subsequent decrease in the extent of cancer cell invasiveness [54]. In fact, our results clearly demonstrated that Ssd is less toxic to human normal lung fibroblast; it could also markedly inhibit the TNF-*α*-induced invasive ability of H1299 human lung cancer cells at nontoxic concentrations, suggesting that Ssd-mediated inhibitory effect on cancer cell invasion is specific, but not due to the direct cytotoxicity of the drug. Besides, COX-2 is implicated in carcinogenic process, and its overexpression by malignant cells is able to enhance cellular invasion, induce angiogenesis, and regulate anti-apoptotic cellular defenses [55]. In addition, VEGF plays multifunctional roles where it may have autocrine prosurvival effect, induce tumor cell chemoresistance [56], and cooperate with mechanical fluid forces to mediate angiogenesis [57]. Therefore, downregulation of both angiogenic factors, COX-2 and VEGF, by Ssd further suggest its anti-angiogenic potential. Besides, recent studies indicate that Ssd inhibits COX-2 in turn to prevent diethylinitrosamine-induced hepatocarcinogenesis in rats, providing an extra evidence for a possible application of Ssd as a chemopreventive agent [58]. Moreover, we adopted the HUVECs tube formation assay using Matrigel to mimic the process of angiogenesis and the results support that Ssd may produce its anti-cancer effect partially through the suppression of angiogenesis. 

 TNF-*α*-induced NF-*κ*B-regulated anti-apoptotic genes including Bcl-2, Bcl-xL, XIAP, c-IAP2, FLIP, and survivin have been correlated with survival, chemoresistance, and radioresistance in numerous tumors [17]. Since these TNF-*α*-induced anti-apoptotic gene products could be significantly downregulated by Ssd treatment, it is believed that Ssd may have potential for further development as an adjuvant agent with TNF-*α* for treatment of chemoresistant tumors. Recent studies provide evidence that Ssd could sensitize cervical and ovarian cancers to cisplatin through ROS-mediated apoptosis [59] and prevent the carcinogen-induced tumorigenesis [58]. Besides, Ssd was further found to potentiate the apoptotic effects of TNF-*α* in our studies. Taken together, Ssd could be utilized as an anti-cancer agent, chemo-preventive agent, or an enhancer for chemotherapy/radiotherapy. In addition, the potency of Ssd in the suppression of NF-*κ*B signaling and its NF-*κ*B-regulated genes expression may be one of the mechanisms against systemic lupus erythematosus [60], pulmonary fibrosis [61], liver inflammation and fibrosis [62], hepatitis B and C [63], viral infection [64], glomerulonephritis [65], and other inflammatory diseases. Cancer is a hyperproliferative disorder characterized by the upregulation of genes responsible for transformation, proliferation, invasion, angiogenesis, and metastasis. Most of these tumorigenic processes are influenced by the aberrant activity of NF-*κ*B signaling. 

## 5. Conclusion

In the current study, we concluded that Ssd effectively inhibits proliferation, suppresses invasion, abrogates angiogenesis, and induces apoptosis in cancer cells through the downregulation of TNF-*α*-mediated NF-*κ*B signaling and its dependent oncogenic genes expression. Most importantly, Ssd displayed low cytotoxic effect on human normal lung fibroblasts. All findings shed light on the potential usage of Ssd as a candidate adjuvant agent together with TNF-*α* for anti-cancer therapy.
